# Auditory feedback decreases timing variability for discontinuous and continuous motor tasks in autistic adults

**DOI:** 10.3389/fnint.2024.1379208

**Published:** 2024-04-16

**Authors:** Nicole Richard Williams, Luc Tremblay, Corene Hurt-Thaut, Jessica Brian, Julia Kowaleski, Kathrin Mertel, Sebastian Shlüter, Michael Thaut

**Affiliations:** ^1^Music and Health Science Research Collaboratory, University of Toronto, Faculty of Music, Toronto, ON, Canada; ^2^College of Music and Performing Arts, Belmont University, Nashville, TN, United States; ^3^Faculty of Kinesiology and Physical Education, University of Toronto, Toronto, ON, Canada; ^4^KITE Research Institute, University Health Network, Toronto, ON, Canada; ^5^Bloorview Research Institute, University of Toronto, Toronto, ON, Canada; ^6^BeSB GmbH Berlin, Sound Engineering, Berlin, Germany; ^7^Faculty of Medicine, Institute of Medical Sciences, University of Toronto, Toronto, ON, Canada

**Keywords:** autism, motor timing, discrete timing, continuous timing, auditory feedback

## Abstract

**Introduction:**

Autistic individuals demonstrate greater variability and timing error in their motor performance than neurotypical individuals, likely due at least in part to atypical cerebellar characteristics and connectivity. These motor difficulties may differentially affect discrete as opposed to continuous movements in autistic individuals. Augmented auditory feedback has the potential to aid motor timing and variability due to intact auditory-motor pathways in autism and high sensitivity in autistic individuals to auditory stimuli.

**Methods:**

This experiment investigated whether there were differences in timing accuracy and variability in autistic adults as a function of task (discontinuous vs. continuous movements) and condition (augmented auditory feedback vs. no auditory feedback) in a synchronization-continuation paradigm. Ten autistic young adults aged 17–27 years of age completed the within-subjects study that involved drawing circles at 800 milliseconds intervals on a touch screen. In the discontinuous task, participants traced a series of discrete circles and paused at the top of each circle for at least 60 milliseconds. In the continuous task, participants traced the circles without pausing. Participants traced circles in either a non-auditory condition, or an auditory condition in which they heard a tone each time that they completed a circle drawing.

**Results:**

Participants had significantly better timing accuracy on the continuous timing task as opposed to the discontinuous task. Timing consistency was significantly higher for tasks performed with auditory feedback.

**Discussion:**

This research reveals that motor difficulties in autistic individuals affect discrete timing tasks more than continuous tasks, and provides evidence that augmented auditory feedback may be able to mitigate some of the timing variability present in autistic persons’ movements. These results provide support for future investigation on the use of music-based therapies involving auditory feedback to address motor dysfunction in autistic individuals.

## Introduction

Autism Spectrum Disorder (ASD, or autism) is a neurodevelopmental disorder which affects 1 in 50 children in Canada, and 1 in 36 children in the U.S. (Centers for Disease Control and Prevention, 2022; Public Health Agency of Canada, 2022). In recent years, many individuals on the autism spectrum have expressed a preference for identity first language (i.e., *autistic person* as opposed to *person with autism*), because autism affects every area of life in someone who is autistic (Taboas et al., 2023). The language used in this article will reflect this majority preference for identity-first language. Autism is characterized by social and communication difficulties and repetitive and restrictive behaviors and patterns of interest, along with sensory sensitivities (American Psychiatric Association, 2013). It is widely acknowledged that atypical motor characteristics and difficulties are also highly prevalent in autistic individuals (Fournier et al., 2010; Bhat et al., 2011; Harrison et al., 2021; Zampella et al., 2021). Increased variability of movements relative to neurotypical individuals along with difficulties in motor timing have emerged as hallmarks of motor difficulties in autism (Torres et al., 2013; Isaksson et al., 2018; Lense et al., 2021). In terms of variability, when attempting the same task (such as a simple reaching motion) multiple times, autistic individuals demonstrate variation in the accuracy of the task completion, such that improvement does not occur in a predictable way over time, as is characteristic for neurotypical individuals (Brinkner and Torres, 2018). Inconsistency in movements can negatively affect a variety of social interactions, sports, and activities of daily living (Brinkner and Torres, 2018). The timing of one’s movements is similarly critical for participation in athletic activities, coordinating movements with others, and reading and expressing body language in social interactions (Bloch et al., 2019; McNaughton and Redcay, 2020; Murat Baldwin et al., 2021). Music-based interventions have strong potential to address motor difficulties in autism like for other neurological conditions (Hardy and Lagasse, 2013; Braun Janzen and Thaut, 2018; Braun Janzen et al., 2022) but underlying mechanisms for music’s effect on movement have not yet been well studied in this population. This study aimed to investigate motor timing and variability in autistic individuals in contrasting movement tasks, and to examine whether augmented auditory feedback could improve motor performance.

It is not yet known whether autistic individuals demonstrate atypical characteristics in timing accuracy and variability differently for discrete versus continuous movements. Discrete movements involve a clear beginning and end, as when clapping hands or touching a screen when dialing a telephone number, while continuous movements do not involve coming to any full stops, as when pedaling while cycling (Huppert and Dyster, 2021). Different timing mechanisms called event and emergent timing are thought to engage depending on whether timing parameters are explicit (in discrete movements) or implicit in the movement (as in continuous movements) (Robertson et al., 1999; Zelaznik et al., 2002). Cerebellar dysfunction can particularly affect the timing and variability of discrete as opposed to continuous movements (Spencer et al., 2003). Differences in cerebellar functioning seem to underpin the difficulties with motor timing and variability in autism to a large extent (Gowen and Miall, 2005; Fatemi et al., 2012; D’Mello and Stoodley, 2015; Mosconi et al., 2015; Oldehinkel et al., 2019; Lidstone et al., 2021). If discrete movements are more difficult for autistic individuals than continuous movements, this may warrant finding strategies for making discrete movements easier or finding alternatives.

An additional aim when researching motor functioning in autism is to identify promising avenues for clinical support of motor skills in autistic children and adults, such as music-based therapies (Braun Janzen and Thaut, 2018). High-quality evidence for interventions that address motor difficulties in autism is increasing but still in the beginning stages of investigation (for reviews, see Colombo-Dougovito and Block, 2019 and Ruggeri et al., 2020). The impact of the sensory system on motor difficulties in autism must be considered when identifying possible interventions. Sensation is intricately connected with online control of movement as well as when building and using internal models, therefore, atypical sensation is associated with atypical movement characteristics in autism (Gowen and Miall, 2005; Robledo et al., 2012; Torres et al., 2013; Lin et al., 2015; Subramanian et al., 2017; Brinkner and Torres, 2018). Some autistic individuals may have 50% fewer intraepidermal nerve fibers than neurotypical children their age (Silva and Schalock, 2016), hypo-or hyper-sensitivities or sensory integration issues (Robledo et al., 2012; Coll et al., 2020), atypical visuo-motor integration (Lidstone and Mostofsky, 2021), and other sensory challenges (Patil and Kaple, 2023). Atypical sensation can be an advantage as well: many autistic individuals show increased sensitivity and brain activity to musical input such as singing and electronic tones (Kuhl et al., 2005; Lepistö et al., 2005; Jones et al., 2009; Sharda et al., 2015). In addition, there is evidence of intact auditory-motor functioning in autistic individuals during rhythmic tapping and perception tasks (Tryfon et al., 2017; Edey et al., 2019; Jamey et al., 2019). Thus, music-based therapies for motor skills in autism have been a growing focus of investigation (Srinivasan and Bhat, 2013; Braun Janzen and Thaut, 2018).

Hardy and Lagasse (2013) and more recently Bharathi et al. (2019) advocated for the use of auditory rhythmic cueing to help regulate movement in autistic individuals. Auditory cueing (like using a metronome) aids with the feedforward control of movement critical in planning and executing movement (Mertel, 2014). Feedforward control involves the interaction of prior internal representations of the motor and sensory components of a movement with sensory feedback to gauge the need for automatic adjustments to movements (Elliott et al., 2010). Voluntary movements involve both feedforward and feedback phases (Elliott et al., 2010). In the feedback phase, movements are controlled online while comparing body position to visual and proprioceptive sensory information (Wolpert et al., 2013). Several studies have found improvements in autistic individuals’ movements with the use of feedforward auditory cues, including in bilateral coordination, balance, running speed and agility, and strength in 8–10-year-old autistic children (El Shemy and El-Sayed, 2018), body coordination (Srinivasan et al., 2015), and increased interpersonal coordination while playing musical instruments to a beat (Kaur et al., 2018; Yoo and Kim, 2018).

The cerebellum is thought to play a major role in the feedforward-feedback interaction process for motor control (Mosconi et al., 2015). Cerebellar dysfunction in autism is thought to impact feedforward control and sensory feedback independently (Mosconi et al., 2015). Thus, to target goal-directed movements more holistically, music-based interventions that focus on providing feedforward information (like a metronome beat) could be complimented by the inclusion of feedback information (sounds from acoustic or digital musical instruments). A handful of studies have investigated the effectiveness of providing auditory feedback alone (i.e., without a metronome) for movement in autistic individuals. Sharda et al. (2018) found that an 8–12-week music therapy intervention using improvisational instrument play (which provides intrinsic auditory feedback) improved auditory-motor neural connectivity in autistic children. Other studies have used augmented auditory feedback to provide electronic auditory stimulation in response to autistic individuals’ movement, with mixed results (Magrini et al., 2015; Wilder et al., 2020). Most recently, Cibrian et al. (2020) tested an auditory-feedback app with 15 autistic children between the ages of 5 and 10. In a set of two studies, Cibrian et al. (2020) found that the children maintained their attention and performed more aimed movements when they were receiving melodic feedback that mirrored the trajectory of the movement (i.e., pitches going up when arms went up). Augmented auditory feedback has helped to improve movement in a variety of clinical populations as well as in neurotypical individuals, and is a promising avenue for clinically-focused research (Fujii et al., 2016; for a review see Schaffert et al., 2019).

Two studies investigated the impact of auditory feedback on timing for discrete and continuous tasks for neurotypical individuals using a task like the one in the current study. Zelaznik and Rosenbaum (2010) added auditory feedback (a “click” upon circle completion at the 12 o’clock point) to a typical synchronization-continuation task with discrete and continuous conditions. They found that the auditory feedback helped decrease mean squared jerk (i.e., improve smoothness of movement) particularly for the discrete movements. Braun Janzen et al. (2021) found that discrete feedback (a tone heard upon circle completion at the 12 o’clock point) improved the coefficient of variation (i.e., decreased timing variability) in both continuous and discontinuous tasks compared to continuous feedback (pitch going down and up as participants traced from the top to bottom of the circle and back up again). Discrete auditory feedback was found effective in promoting better motor timing in both studies (Zelaznik and Rosenbaum, 2010; Braun Janzen et al., 2021).

This study thus aimed to assess the effect of auditory feedback on timing error and consistency in discrete and continuous tasks in autistic adults. Two research questions were posed: (1) Will the task (continuous or discontinuous) have a significant effect on timing error or variability across conditions? (2) Will the condition (with or without auditory feedback) have a significant effect on timing error or variability across discontinuous and continuous movement tasks?

## Methods

The study was designed by the first author in consultation with her dissertation committee, and the experimenters were two PhD students who were also neurologic music therapists with experience working with autistic individuals and those with developmental disabilities. The study was approved by the University of Toronto Research Ethics Board.

### Participants

Twelve young adults between the ages of 17 and 27 years (mean = 22 years) who confirmed in writing that they had received an official autism diagnosis participated in the study. Intelligence quotient (IQ) was not measured as a part of the study. All participants possessed the written and verbal communication skills to independently communicate their interest in the study via email, schedule a date and time, navigate to the lab where the experiment took place, and communicate verbally with the experimenter during the experiment. Participants were recruited in the Greater Toronto Area, Canada using a combination of paper advertisements on the University of Toronto St. George campus, online advertisements, and the sharing of posters on social media. Each participant was provided with a detailed informed consent form with information about the study and signed their consent. To be included in the study, participants were required to have normal or corrected-to-normal vision, no hearing problems, no problems with motor skills in daily life, and to not be professional musicians or athletes (defined as having at least 10 years of experience up to the current year playing an instrument or playing a competitive sport) (Braun Janzen et al., 2014). All participants fit these criteria except for one participant with partial hearing loss in both ears and some vision loss in one eye. That participant was able to adjust the headphone volume to be adequate and could carry out the visual aspects of the experiment. The questionnaire collecting demographic data used open-ended questions (i.e., “What is your race/ethnicity?,” “What is your gender?”; see Table 1). Almost half of participants (41.7%) identified as non-binary or trans in the current study, which is commensurate with recent research by Corbett et al. (2023) who found that autistic individuals are far more likely to identify as non-binary than neurotypical peers.

**Table 1:** Demographic information.

Number of participants (*N*)	12
Participants with complete data (*n*)	10
	*Mean (SD)*
Age range: 17–27	22 (4.4)
	*N*
Participants with additional diagnoses (most commonly: attention deficit hyperactivity disorder; major depressive disorder, anxiety disorders, obsessive-compulsive disorder).	12
Participants taking at least one medication	9
Participants who reported having at least one sensory sensitivity	7
Gender	
Male	4
Female	3
Non-binary	4
Trans	1
Handedness	
Right	12
Race/Ethnicity	
White	5
Mixed	3
Black	1
South Asian	1
Han Chinese	1
Not disclosed	1

Due to experimenter error, data from two participants were collected with a different auditory feedback condition, so these data were not included in the main analysis. A separate analysis of the data from these two participants is included at the end of the results section. Of the remaining 10 participants, one participant completed three of the four conditions, and data from one of four conditions from a different participant was lost due to equipment failure. Each of the other eight participants completed all four conditions.

### Materials

#### Apparatus

Participants sat on a chair at a comfortable distance from a 25-inch tall table on which sat the touch-screen device which was inclined towards participants at approximately 10 degrees. A custom-built device running custom software (SONification Arm Training Apparatus [SONATA] by BeSB GmbH Berlin) was used to present stimuli and collect data (Schaffert et al., 2020). Software was identical to that used in Braun Janzen et al. (2021). The SONATA device consisted of a custom-made graphical user interface generated by a single-board computer (Raspberry Pi 2 B with HiFiBerry). Software output was displayed on a 32″ touchscreen device (AG Neovo TX-32P, resolution: 1920 × 1080 pixels), and the built-in sound-output was funneled to participants via noise-cancelling headphones plugged into a headphone splitter inserted into the touchscreen. Noise-cancelling headphones were not available for two participants due to equipment failure, and earbud-style headphones were used for those two participants. Latency was approximately <30ms from the moment of touching the screen to sound output.

### Tasks and procedure

The experiment used a synchronization-continuation paradigm (Stevens, 1886). Although beat synchronization is sometimes more variable in autism (Kaur et al., 2018; Gabis et al., 2020; Franich et al., 2021), there is evidence that auditory synchronization in autistic individuals is not dissimilar on average to that of neurotypical individuals, particularly for self-timed movements (Tryfon et al., 2017; Vishne et al., 2023), indicating that a synchronization-continuation paradigm is appropriate for autistic adults. The experiment protocol was piloted with two neurotypical adults and one autistic adult to ensure the task was reasonable.

Each participant carried out the experimental protocol in one session. At the beginning of the session, participants signed the informed consent and intake question forms and were provided with instructions on how to perform the tasks. The experiment consisted of two blocks of twenty trials (continuous and discontinuous tasks), divided by condition (10 trials with augmented auditory feedback and 10 without augmented auditory feedback) (Braun Janzen et al., 2021). This experimental design thus yielded four task-condition combinations: Continuous circles with augmented auditory feedback, continuous circles without auditory feedback, discontinuous circles with augmented auditory feedback, and discontinuous circles without auditory feedback (Spencer et al., 2003; Zelaznik and Rosenbaum, 2010). The order of tasks was counterbalanced across participants, as was the order of conditions within each task block. Participants could practice the task before each experiment block for up to ten trials. Most did not require a full set of ten practice trials before they felt ready to commence the experiment. If needed, the experimenter demonstrated the task. Participants could adjust their headphone volume to their preferred level. They could take a break whenever they wished between trials or blocks, and sensory items such as a fidget spinner, playdough, and a squish toy were available if helpful to participants when taking a break.

**Figure 1:**
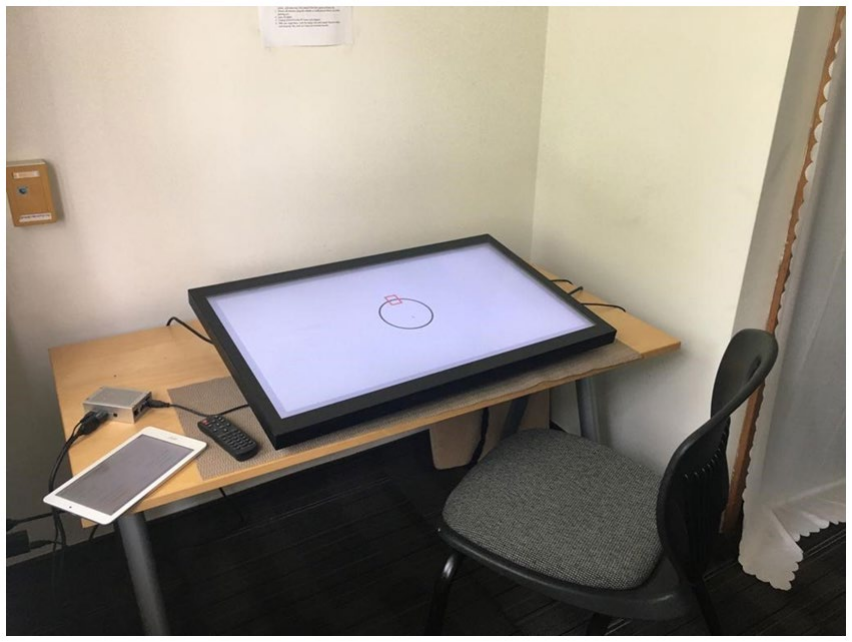
Physical setup of the task.

Participants were instructed to trace a circle with the index finger of their dominant hand, ensuring their finger passed over the target mark (a rectangle at the 12 o’clock point in the circle) in synchrony with an isochronous metronome click at an isochronous 800 millisecond (ms) interval (General MIDI sound set, channel 10, MIDI Key 60, “High Bongo” percussion timbre). For each trial, after 10 cycles of the synchronization phase in all conditions and tasks, the metronome stopped, and participants were instructed to continue their movements at the same tempo (to the best of their ability) until the end of the trial. The continuation phase involved 20 additional drawing cycles, for a total of 30 circles per trial. The circle they traced was 10.5 cm (about 4.13 in) in diameter. Participants were told that timing was more important than spatial accuracy, so circles they drew did not have to exactly match the circle template. Participants could see their hands, and their movements generated traces on the screen creating visual feedback of their movement trajectories. Movements were observed to be executed mainly via elbow and shoulder motion. Participants had to keep their finger on the touchscreen for the duration of each trial. If a finger came off the screen prematurely, the trial automatically aborted and had to be restarted (see Figure 1).

In the auditory conditions, augmented auditory feedback (General MIDI sound set, channel 80, “Square Lead”) was triggered whenever participants crossed their finger into the target rectangle at the top of the circle during both the synchronization and continuation phases. In the non-auditory trials, auditory feedback was not provided in the continuous phase. Because the software was designed to provide auditory feedback in all conditions, the conditions without auditory feedback were created manually by the experimenters unplugging the participant’s headphones from the splitter after the 10 metronome clicks in the synchronization phase were completed. Participants were told whether or not they would receive auditory feedback, at the beginning of each set of trials.

**Figure 2:**
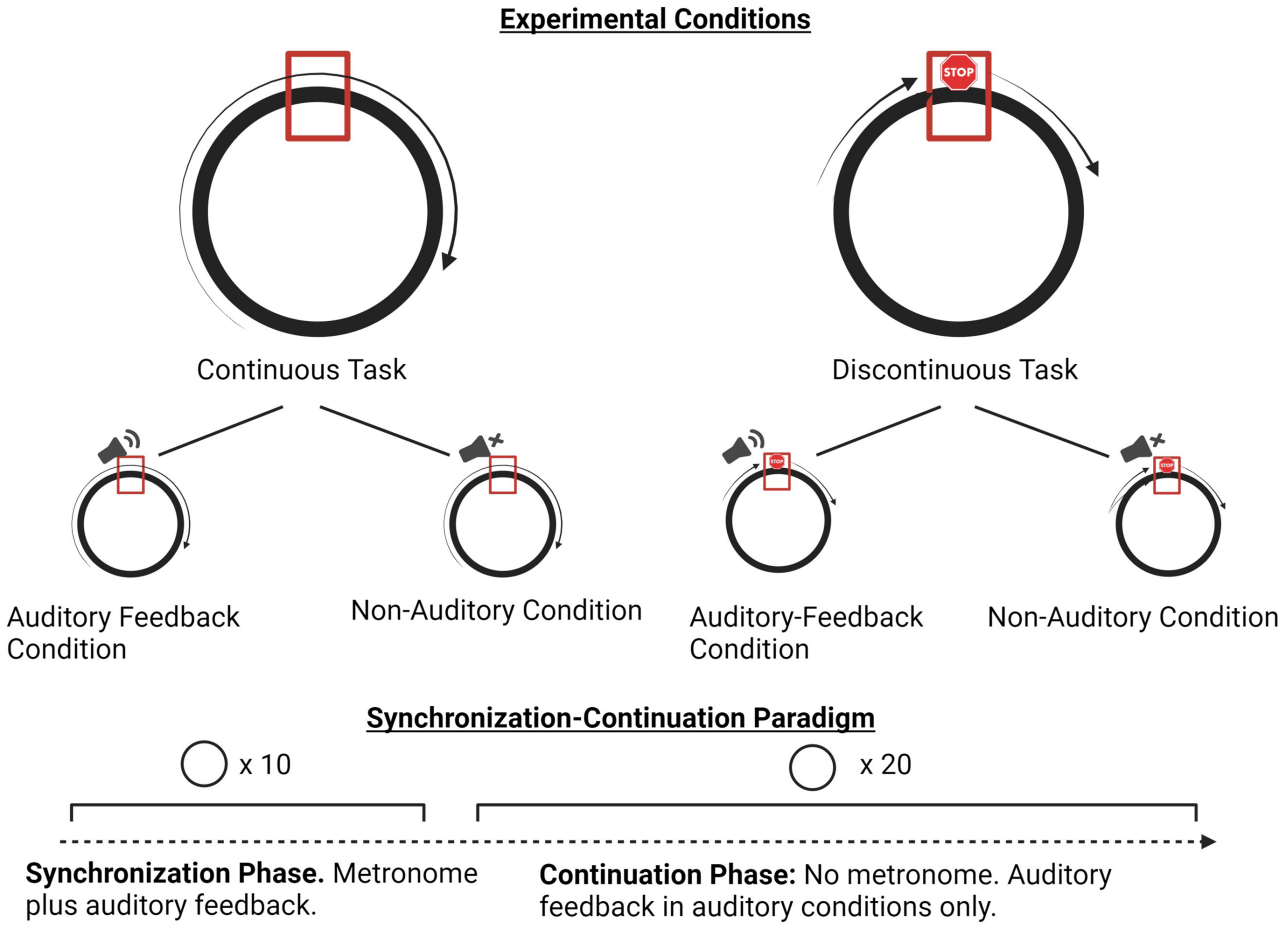
Overview of experiment paradigm and conditions. Created with BioRender.com.

In the continuous circle drawing condition, participants were instructed to complete the drawing cycles in a continuous motion and aim to pass through the rectangle at the top of the circle in conjunction with the metronome clicks, continuing with this cycle after the metronome ended and until the trial ended as indicated on the screen. In the discontinuous circle drawing task, participants were instructed to briefly pause their finger motion on the rectangle target at the top of the circle once per drawing cycle, rendering the task a series of discrete circles. The software was designed such that if the participants stopped for less than 60 ms, the trial automatically ended and restarted. Note that the software did not directly provide information on the velocity of the participants’ finger motions within the target area, in which they had to spend a minimum of 60 ms. Thus, it is not possible to calculate a precise dwell time (Adam and Paas, 1996). Typically, movements in a series can be considered discrete only when movement slows to near-zero velocity, indicating the beginning or end of a movement (Hogan and Sternad, 2007). Participants spent upwards of 156 ms in the (small) target area, and were observed to pause their movements in between iterations of the circle. For these reasons, the study is interpreting the movements in the discontinuous circle drawing task as a series of discrete movements, as in Braun Janzen et al. (2021). Figure 2 provides a visual representation of the tasks and paradigm.

After completing the experiment, participants filled out and signed a brief exit survey which included an opportunity to consent to participate in future studies and were invited to choose one $15 CAD gift card to thank them for participating. The procedure took approximately one hour to be completed.

### Data analysis

The continuation phase of each trial was measured in terms of twenty inter-response intervals (IRI) between each entry of the target area, measured in milliseconds (ms). The mean, standard deviation, standard error of the mean, and coefficient of variation for the IRIs were calculated for each trial, and then averaged across the best trials within each task and condition. Mean IRI was considered a measurement of timing accuracy. Means closer to 800 ms (the tempo of the metronome during the synchronization phase) indicated better timing accuracy. Coefficient of variation (CV) was the measure of timing variability. CV equals the IRI standard deviation divided by the IRI mean, converted to a percentage. If the standard deviation was large compared to the mean, this yielded a higher CV, indicating more variability in the IRI over repetitions of the circle drawing within a trial. Conversely, a smaller CV indicated lower variability of movement timing.

The best eight trials (determined by the lowest eight values for coefficient of variation [CV]) within each condition for each participant were included in the analysis (Braun Janzen et al., 2021). Only data from the continuation phase was used in the analysis as the synchronization phase was used for pacing only.

Two-way repeated-measures ANOVAs were performed on the mean IRI and mean CV to compare timing accuracy and variability between tasks (continuous vs. discontinuous) and conditions (with auditory feedback vs. without auditory feedback). The assumption of normality was satisfied for most categories in both ANOVAs. Departures from normality were tested and found via a Shapiro–Wilk test in one of each of the four categories in mean IRI data (*p* = 0.00005) and in the CV data (*p* = 0.02), respectively. Observation of Q-Q plots indicated that these departures from normality were due to some outliers in the data. Because the outliers were due to the natural variation in motor characteristics between autistic participants, they were left in the data. ANOVAs are typically robust to violations of the assumption of normality when the assumption of sphericity is met, as was automatically the case for this data since each factor had only two levels (Blanca et al., 2023).

### Additional analysis

Two participants were mistakenly given experimental tasks using a software setting that provided continuous auditory feedback as they traced around the circle rather than the standard simple tone provided only at the top of the circle. Data from the two participants in the current study who received the continuous auditory feedback were excluded from the main analysis, but the mean IRI and CV data was analyzed descriptively.

### Exit survey

The exit survey contained two questions: The first was *How easy or hard did you find this experiment?* with a five-point Likert scale of *Easy, Somewhat Easy, Neither Easy nor Hard, Somewhat Hard, and Hard.* The second was: *Do you have anything you’d like to tell us about your experience?* Participants were also asked if they consented to be contacted for future studies.

## Results

**Figure 3:**
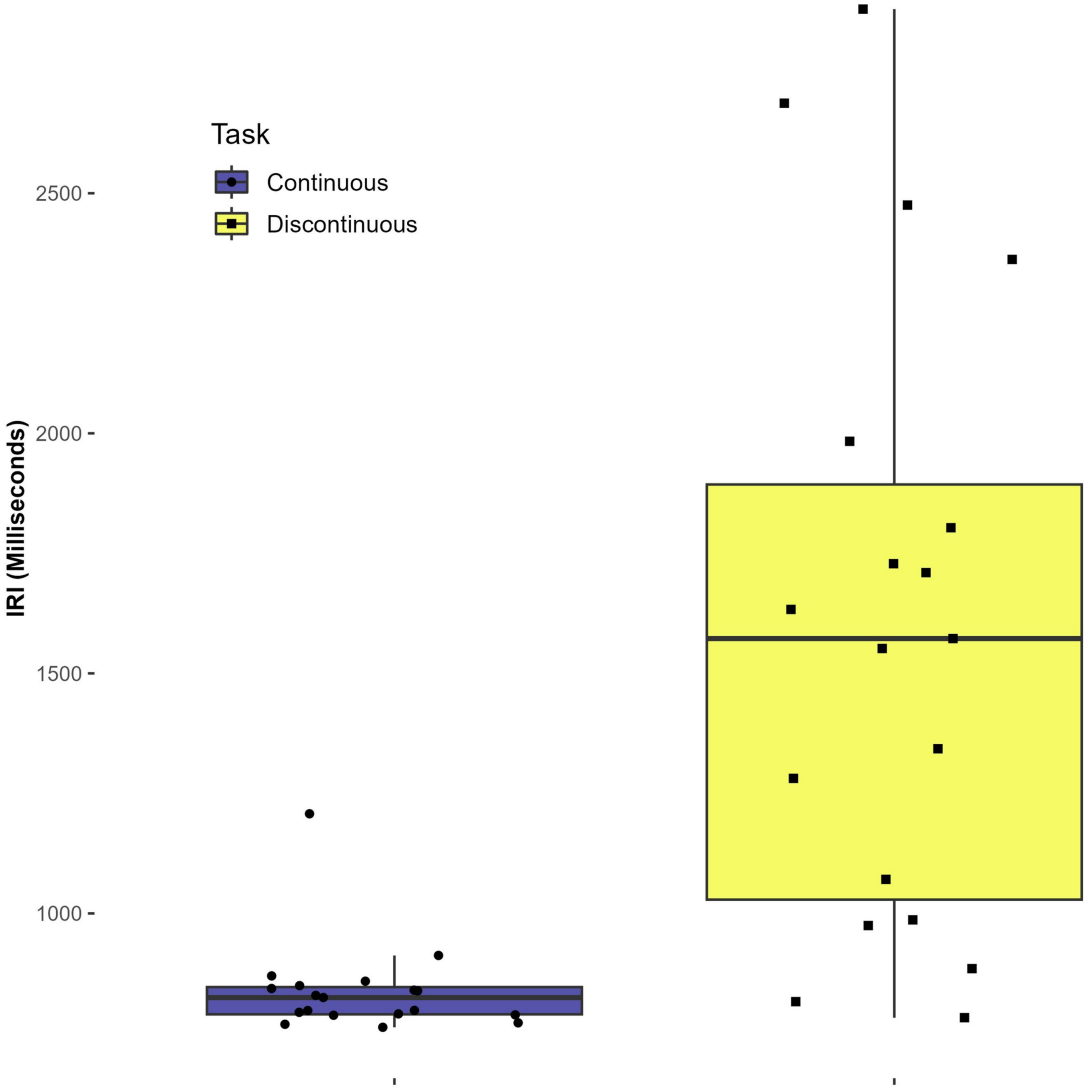
Effect of task on mean inter-response interval (IRI).

There was a statistically significant main effect of task on the mean IRI, *F*(1,7) = 10.79, *p* = 0.013, η^2^ = 0.380. The mean IRI for the continuous task was 839 ms (SD = 97) while the mean IRI for the discontinuous task was 1607 ms (SD = 641). The main effect indicates that participants took a significantly longer time during the discontinuous task across conditions. No other statistically significant main or interaction effects were found in the mean IRI analysis (*p* > 0.3). See Figure 3.

**Figure 4:**
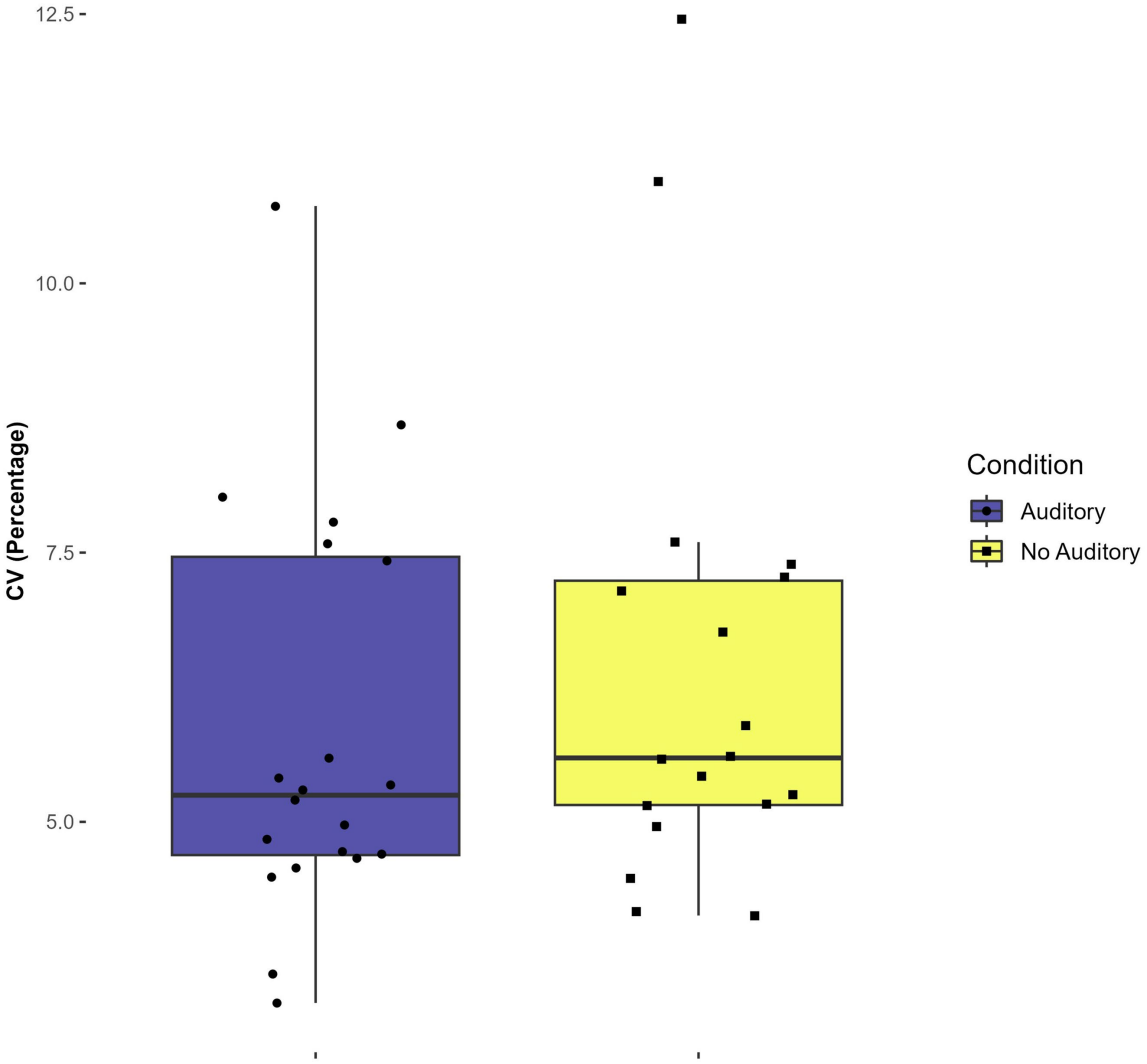
Effect of auditory condition on mean coefficient of variation (CV).

There was a statistically significant main effect of condition (with auditory feedback vs. without) on the CV, *F*(1,7) = 6.149, *p* = 0.04, η^2^ = 0.045. The mean CV for the condition with auditory feedback was 5.9 percent (SD = 1.9) while the mean CV for the conditions with no auditory feedback was 6.4 percent (SD = 2.2). This result indicates that participants had lower timing variability when hearing auditory feedback at the completion of each circle versus when receiving no auditory feedback, when averaged across tasks. No other significant main or interaction effects were found in the CV analysis (*p* > 0.9). See Figure 4.

### Strategies in the discontinuous task

Because the mean IRI was significantly larger for the discontinuous task compared to the continuous task, an additional analysis was conducted to ascertain what strategy participants were using to complete the discontinuous task. Mean dwell time for each participant (defined as the number of milliseconds spent in the rectangle target area at the top of the circle) was calculated and compared to the mean IRI. These values were calculated for the discontinuous trials in the continuation phase only. Participants diverged in the strategies used to complete the discontinuous circle task. A few spent relatively little time (100-300ms) in the target area compared to the minimum of 60 ms, and had IRI values closer to 800 ms than other participants. Others took the strategy of spending half of the time in the target rectangle, and the other half moving around the circle. This yielded an IRI of around 1600 ms. The other participants had no discernable strategy – they simply took longer to complete each circle and spent 300–700 ms in the target rectangle on average. Because the mean IRI for the discontinuous task was 1606.96 ms (SD = 641.43 ms), this could falsely indicate that most participants utilized the strategy of spending half of their time in the target rectangle, so it is important to clarify that a wide range of strategies were utilized. Though it is possible that some individuals employed different spatial trajectories along with timing strategies, differences in spatial strategies were not measured. See Table 2.

**Table 2:** Discontinuous trial times and strategies.

Apparent strategy	Discontinuous trial, with auditory feedback			Discontinuous trial, without auditory feedback			Participant	Mean (SD) IRI (ms)	Time in target (ms)	% of time in target	Mean (SD) IRI (ms)	Time in target (ms)	% of time spent in target
Aiming for 800 ms IRI	783 (36)	171	22.0	816 (44)	186	22.9	P10
Aiming for 800 ms IRI	987 (55)	180	17.7	885 (47)	156	17.1	P6
Aiming for 800 ms IRI	1071 (48)	275	25.6	975 (41)	349	34.6	P2
Aiming for 800 ms IRI	1343 (73)	384	28.9	1281 (94)	354	27.1	P4
Taking more time	1710 (127)	562	32.6	2779 (329)	797	30.2	P8
Taking more time	1552 (116)	589	38.2	1803 (102)	540	29.9	P9
Taking more time	1984 (155)	618	32.0	2476 (190)	449	19.4	P3
Half target, half circle	1633 (58)	760	46.4	1573 (65)	787	49.6	P5
Half target, half circle	1762 (130)	940	55.0	NA	NA	NA	P1
Hybrid between taking more time and half target/half circle (1/3 target, 2/3 circle).	2362 (256)	992	40.5	2713 (269)	977	32.9	P7

### Additional analysis with continuous auditory feedback

**Figure 5:**
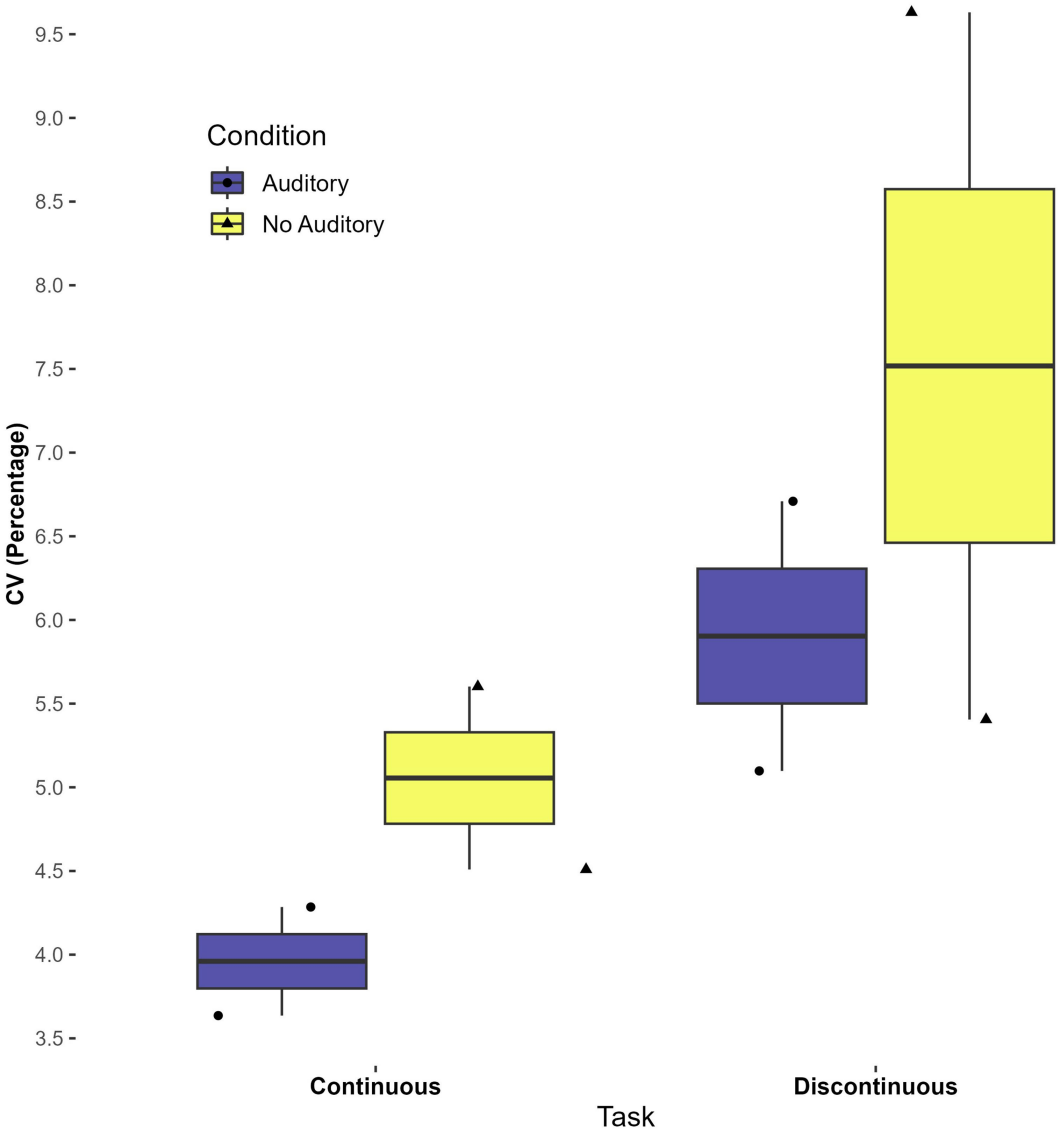
Differences in mean CV across tasks and conditions with continuous auditory feedback.

**Figure 6:**
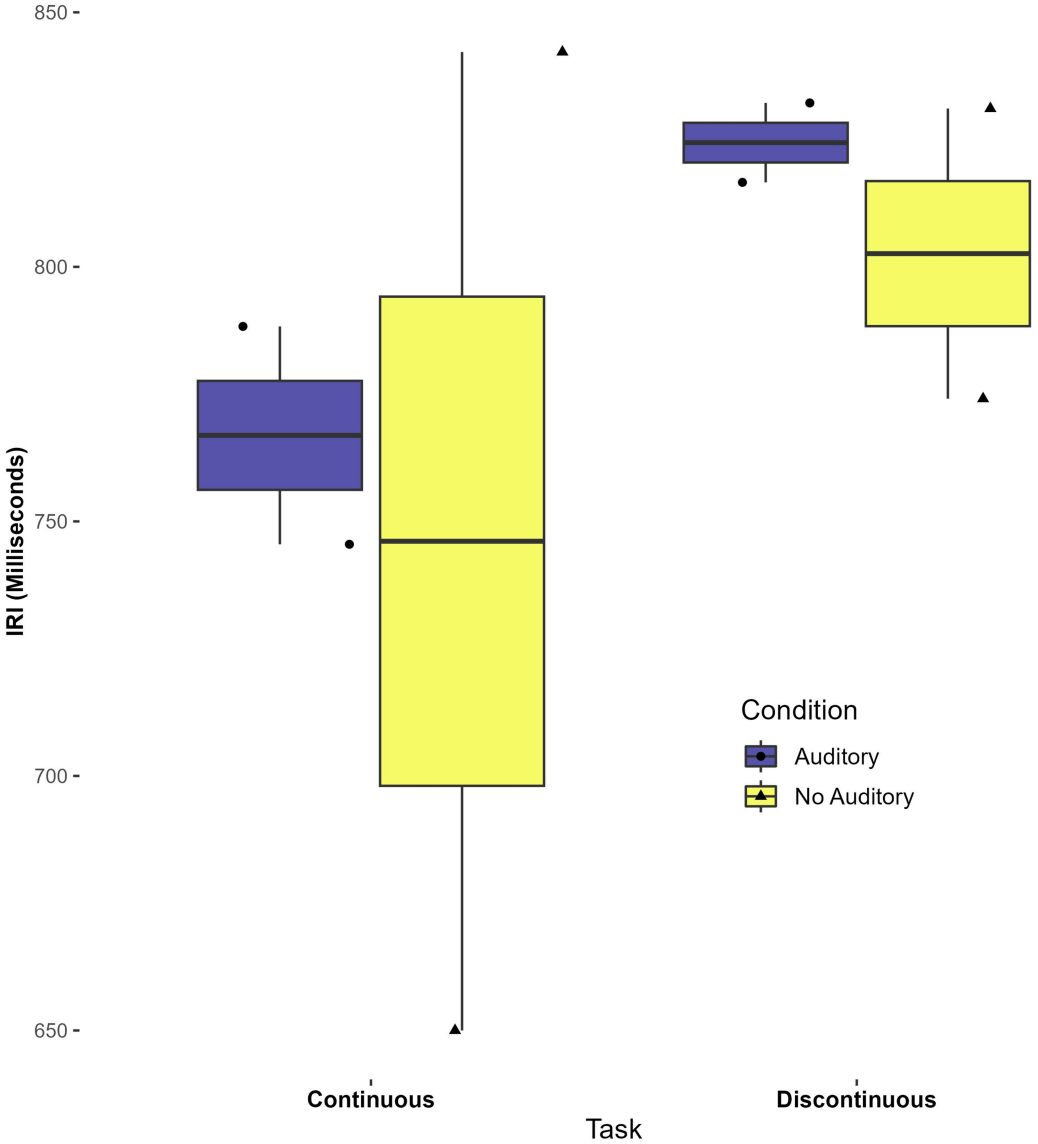
Differences in mean IRI across tasks and conditions with continuous auditory feedback.

Though the current study did not aim to investigate whether perceptual-motor matching aided timing (Braun Janzen et al., 2021), a preliminary investigation was done using continuous auditory feedback with two participants. Boxplots indicate that the CV was lower (i.e., lower variability) in conditions with continuous auditory feedback, for both continuous and discontinuous tasks. Interestingly, average IRI appeared to be more consistent (a narrower spread) across participants in the auditory task. These findings, although with a very small sample, suggest that continuous auditory feedback merits further investigation in autistic participants as an aid to motor timing. See Figures 5, 6.

### Exit survey results

**Figure 7:**
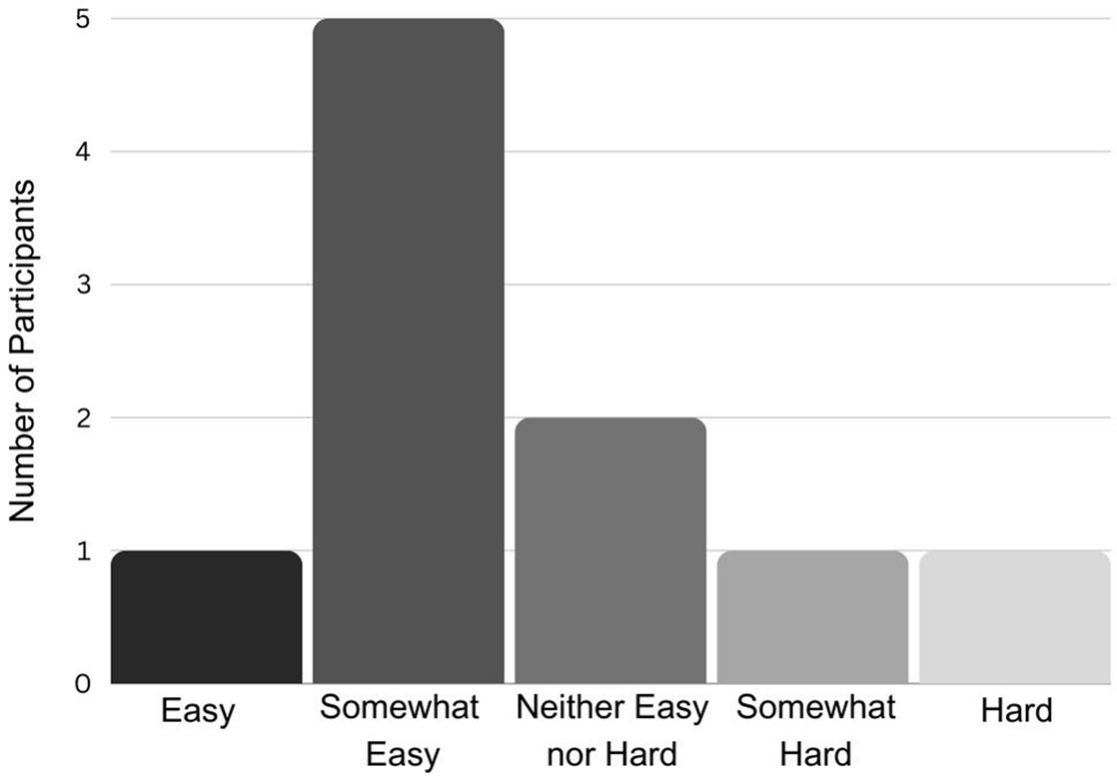
Participant perceptions regarding difficulty of the experiment.

Most participants reported that the experiment was easy or somewhat easy (Figure 7). Responses to the question “Do you have anything you’d like to tell us about your experience?” were varied. Five participants left no comment. One participant reported: “I think the background noise from the practice rooms might have had an impact on my internal metronome-ing!” The experimental room was in a music building with practice rooms above, and the sound of pianists practicing could sometimes be heard in the room. It is possible that despite noise-cancelling headphones, the sound of the piano distracted some participants, though only one participant out of ten reported being distracted. Other participants reflected on the process of doing the experiment, stating: “I noticed when I did not have audio, my circles were circles. When I did, I’d pick the opposite point, go up and down. I do not know why”; “I had to pay attention to my technique even within the same trial. I adjusted my rhythm even within the same trial”; “Was interesting but a bit nervewracking [*sic*]. It was a bit vague from the instructions but I was able to get the hang of it quickly.” One participant simply wrote, “It was fun.” In general, most participants seemed to enjoy being part of the experiment, were curious about the general topic (motor skills in autism) and many requested (with affirmative response from the researcher) that a copy of the published results be shared with them.

## Discussion

### Task results

Participants in this study struggled more with their timing accuracy in the discontinuous circle-drawing than in the continuous circle-drawing task. The pattern of increased difficulty in the discontinuous task is similar to the results seen in Spencer et al.’s (2003) research with individuals with cerebellar lesions, who performed significantly more poorly on a discontinuous task than a continuous task. The current study’s results thus do not contradict research indicating the presence of cerebellar dysfunction in autism (Fatemi et al., 2012).

### Implications of task results

The finding that the discontinuous circle task was more challenging for the participants in this study also echoes findings of Fabbri-Destro et al. (2009), who found that autistic individuals (in their case, children) struggled to chain multiple discrete actions together. Having difficulty chaining actions has real-world implications for movement tasks required of autistic individuals. For example, some autistic persons use augmentative and alternative communication (AAC) approaches to aid with interaction (Aydin and Diken, 2020). AAC strategies can include Picture Exchange Communication Systems in which the individual hands picture-cards representing needs or wants to a person who can assist them, and Speech Generating Devices in which the individual uses a touch-screen device pre-programmed with various words to communicate needs, which are then translated into electronic auditory speech by the device (Aydin and Diken, 2020). Other autistic individuals have found means of expression using keyboards or computers and independently typed their words. Importantly, both Speech Generating Devices and typing (when done with one finger) require a chain of discontinuous movements. Because continuous movements may be much simpler for autistic individuals, perhaps communication devices involving continuous motions rather than discontinuous motions could be developed for use by non-speaking autistic individuals. These devices could work with the strengths of autistic individuals’ motor systems (continuous motions) rather than trying to fit their communication using devices designed for the neurotypical motor system.

### Condition results

Autistic participants performed with greater overall consistency (lower coefficient of variation) when they received discrete auditory feedback at the completion of each circle. This result was observed in both continuous and discontinuous conditions. Autistic adults rely more on auditory feedback for speech production as they experience more significant disruptions in speech than neurotypical adults when the sound of their speech in headphones is slightly delayed (Lin et al., 2015). Previous studies by Cibrian et al. (2020), Magrini et al. (2015), and Wilder et al. (2020) reported that software providing augmented auditory feedback is engaging and may improve some aspects of movement. The current study adds to this literature and explicitly demonstrates that auditory feedback can improve autistic individuals’ performance on motor timing.

Research on the use of auditory feedback is important in the clinical world because neurologic music therapy interventions such as Therapeutic Instrumental Music Performance (TIMP)^®^ utilize both feedforward auditory stimulation (a metronome) and task-intrinsic auditory feedback (the sound of an instrument playing). However, reviews and preliminary studies on the use of music for movement in autism (aside from Cibrian et al., 2020) have largely focused on the potential of rhythmic auditory cueing alone as the main driver for change without examining the potential of auditory feedback (Hardy and Lagasse, 2013; El Shemy and El-Sayed, 2018). The current study supports the use and further research of auditory feedback to address motor timing in autistic individuals, and provides justification for future clinical studies of TIMP to address motor skills in autistic individuals. A pilot study conducted by the first author (under review) found that the motor skills of autistic children improved after a three-week, nine-session Neurologic Music Therapy (NMT)^®^ intervention largely employing TIMP to address motor skills. More studies should continue to investigate the effects of intrinsic and augmented auditory feedback both independently and when used with rhythmic auditory cueing on motor functioning in autistic individuals.

Because timing consistency seems to be one of the functions of the cerebellum (Spencer et al., 2003), it is interesting that auditory feedback improved the coefficient of variation in the current study, given that autistic individuals are generally thought to experience cerebellar dysfunction (Fatemi et al., 2012). Future studies could examine by what mechanism auditory feedback allows for improvements in motor timing consistency.

### Limitations

The main limitation of the current study is its small sample. It would additionally have been interesting to compare results with autistic adults to a control group of neurotypical participants, particularly regarding the performance on the continuous vs. discontinuous tasks. While like patients with cerebellar lesions, autistic adults struggled more with the discontinuous tasks than the continuous tasks, it is unknown whether their performance on continuous tasks is statistically different than that of neurotypical adults. A comparison group of patients with cerebellar lesions would provide additional information and insight regarding any similarities in performance between neurotypical and autistic adults and patients with cerebellar dysfunction.

Though the auditory feedback provided a statistically significant reduction in coefficient of variation, the effect size was small. More research is needed to measure whether the effects of auditory feedback can improve motor variability on motor tasks of everyday life that may be of interest to individuals in the autism community.

## Conclusion

Autistic adults struggled more with discrete timing tasks than continuous timing tasks. Autistic adults can leverage augmented auditory feedback without the presence of a concurrent rhythmic auditory cue to decrease their movement variability at a precise level for continuous and discontinuous timing tasks. This paper provides support for the potential of music-based motor rehabilitation techniques involving augmented auditory feedback to decrease motor variability in autistic persons. More research is needed to ascertain the clinical potential of auditory feedback to improve various motor parameters to an extent that is meaningful for the everyday life experiences and needs of autistic individuals.

## Data availability statement

The raw data supporting the conclusions of this article will be made available by the authors, without undue reservation.

## Ethics statement

The studies involving humans were approved by University of Toronto Research Ethics Board. The studies were conducted in accordance with the local legislation and institutional requirements. The participants provided their written informed consent to participate in this study.

## Author contributions

NRW: Conceptualization, Formal analysis, Investigation, Methodology, Project administration, Visualization, Writing – original draft, Resources, Writing – review & editing. LT: Supervision, Writing – review & editing. CH-T: Supervision, Writing – review & editing. JB: Writing – review & editing, Supervision. JK: Investigation, Writing – review & editing. KM: Investigation, Writing – review & editing. SS: Software, Writing – review & editing. MT: Supervision, Writing – review & editing.
